# Supplementary material for: miRNA142-3p targets Tet2 and impairs Treg differentiation and stability in models of type 1 diabetes
Source: Nat Commun. 2019 Dec 13;10:5697. doi: 10.1038/s41467-019-13587-3 (PMC6910913; doi:10.1038/s41467-019-13587-3)
Supplement: Supplementary file 1 — Supplementary Material [file 41467_2019_13587_MOESM1_ESM.pdf]

Supplementary Information for:

miRNA142-3p targets Tet2 and impairs Treg differentiation and stability in models of type 1 diabetes

Scherm et al.

This PDF file includes: Supplementary Figures 1-17, Table S1

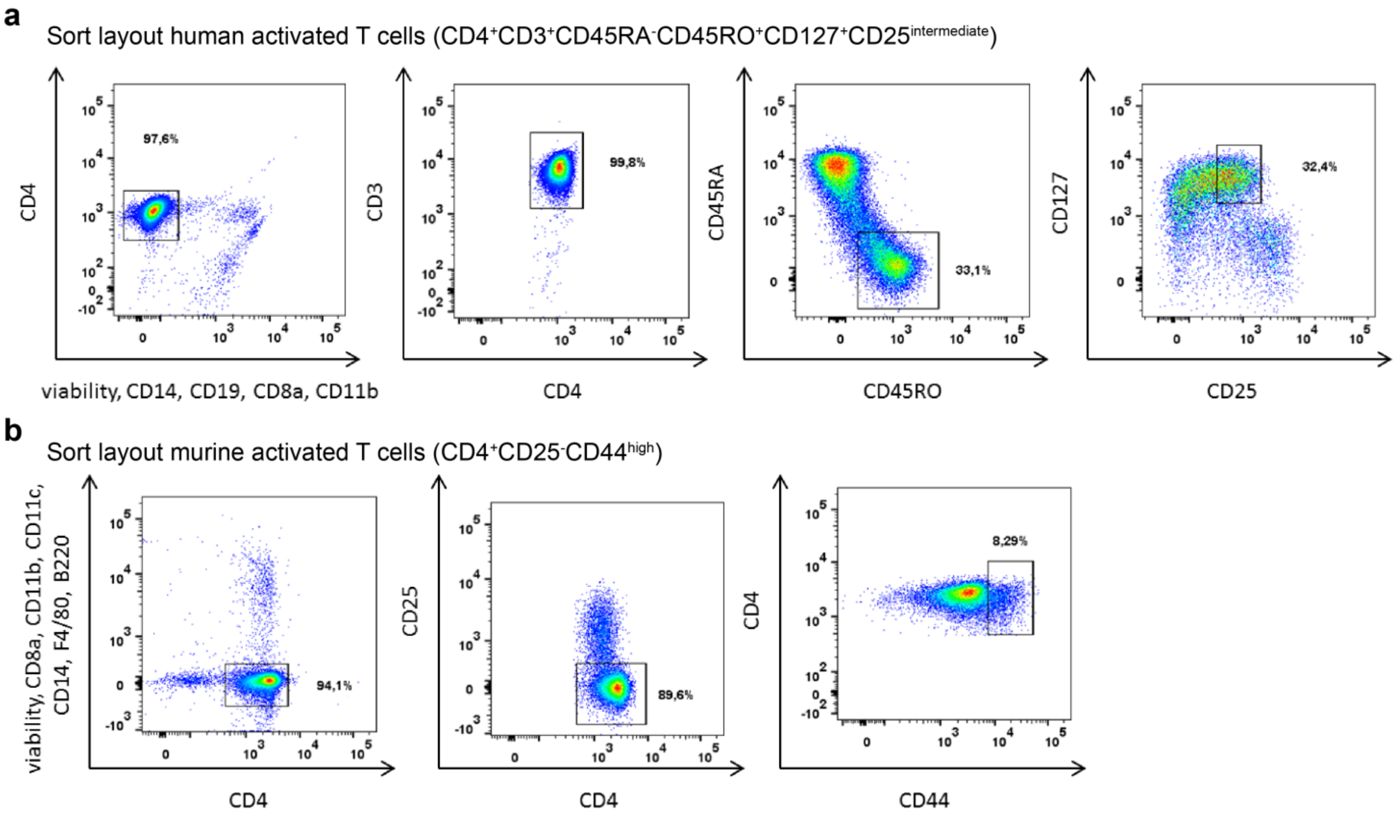

Supplementary Figure 1. Gating strategy for FACS sorting of human and murine activated T cells. (a) Representative FACS plot for the sorting of human activated T cells: $CD4^{+}CD3^{+}CD45RA^{-}CD45RO^{+}CD127^{+}CD25^{intermediate}$. **(b)** Representative FACS plot for the sorting of murine activated T cells: $CD4^{+}CD25^{-}CD44^{high}$.

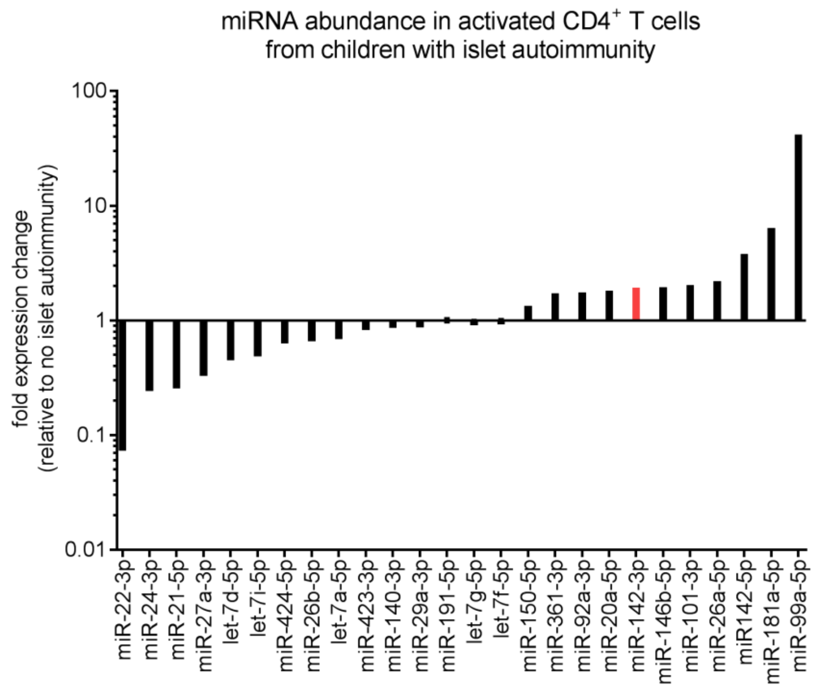

Supplementary Figure 2. Changed miRNA abundance in activated CD4$^+$ T cells from children with ongoing islet autoimmunity. Fold expression change of miRNAs in activated CD4$^+$ T cells from children with ongoing islet autoimmunity compared to healthy controls.

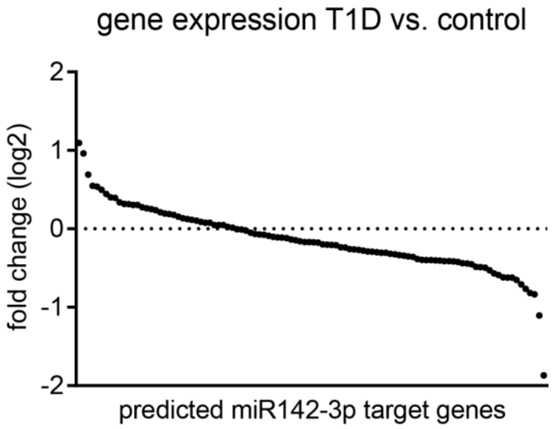

Supplementary Figure 3. Predicted miR142-3p targets are downregulated in CD4$^+$ T cells from individuals with ongoing islet autoimmunity. Fold expression change of 104 predicted miR142-3p target genes in CD4$^+$ T cells from individuals with ongoing islet autoimmunity compared to healthy controls. Source data are provided as a Source Data file.

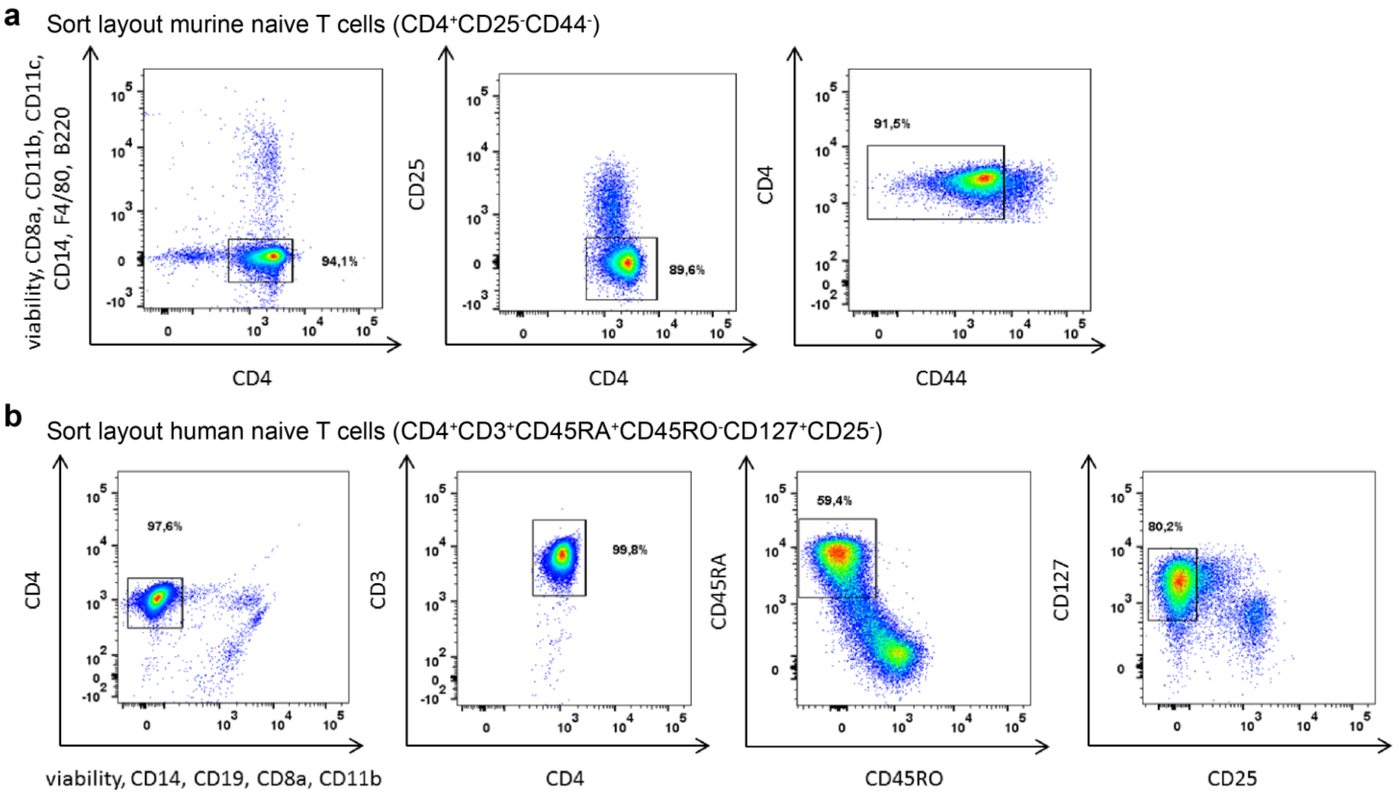

Supplementary Figure 4. Gating strategy for FACS sorting of murine and human naive T cells. (a) Representative FACS plot for the sorting of murine naive T cells: $CD4^{+}CD25^{-}CD44^{-}$. **(b)** Representative FACS plot for the sorting of human naive T cells: $CD4^{+}CD3^{+}CD45RA^{+}CD45RO^{-}CD127^{+}CD25^{-}$.

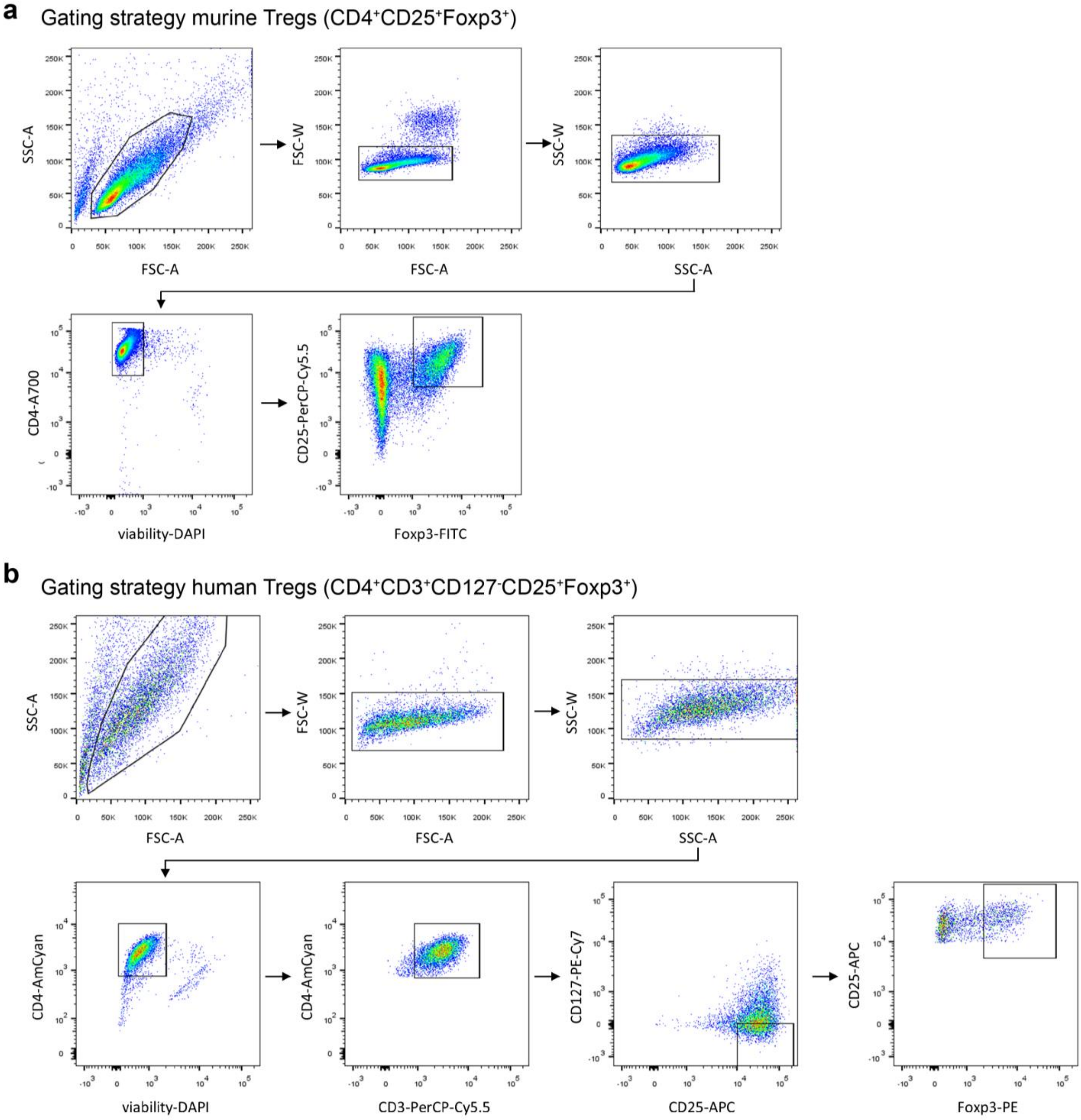

Supplementary Figure 5. Gating strategy for FACS sorting of induced murine and human Treg. (a) Representative FACS plot for the sorting of induced murine Tregs: $CD4^{+}CD25^{+}Foxp3^{+}$. These gating panels correspond to Figures 2a, c-f and 5i. **(b)** Representative FACS plot for the sorting of induced human Tregs: $CD4^{+}CD3^{+}CD127^{-}CD25^{+}Foxp3^{+}$. These gating panels correspond to Figures 3a, c-e and 5j.

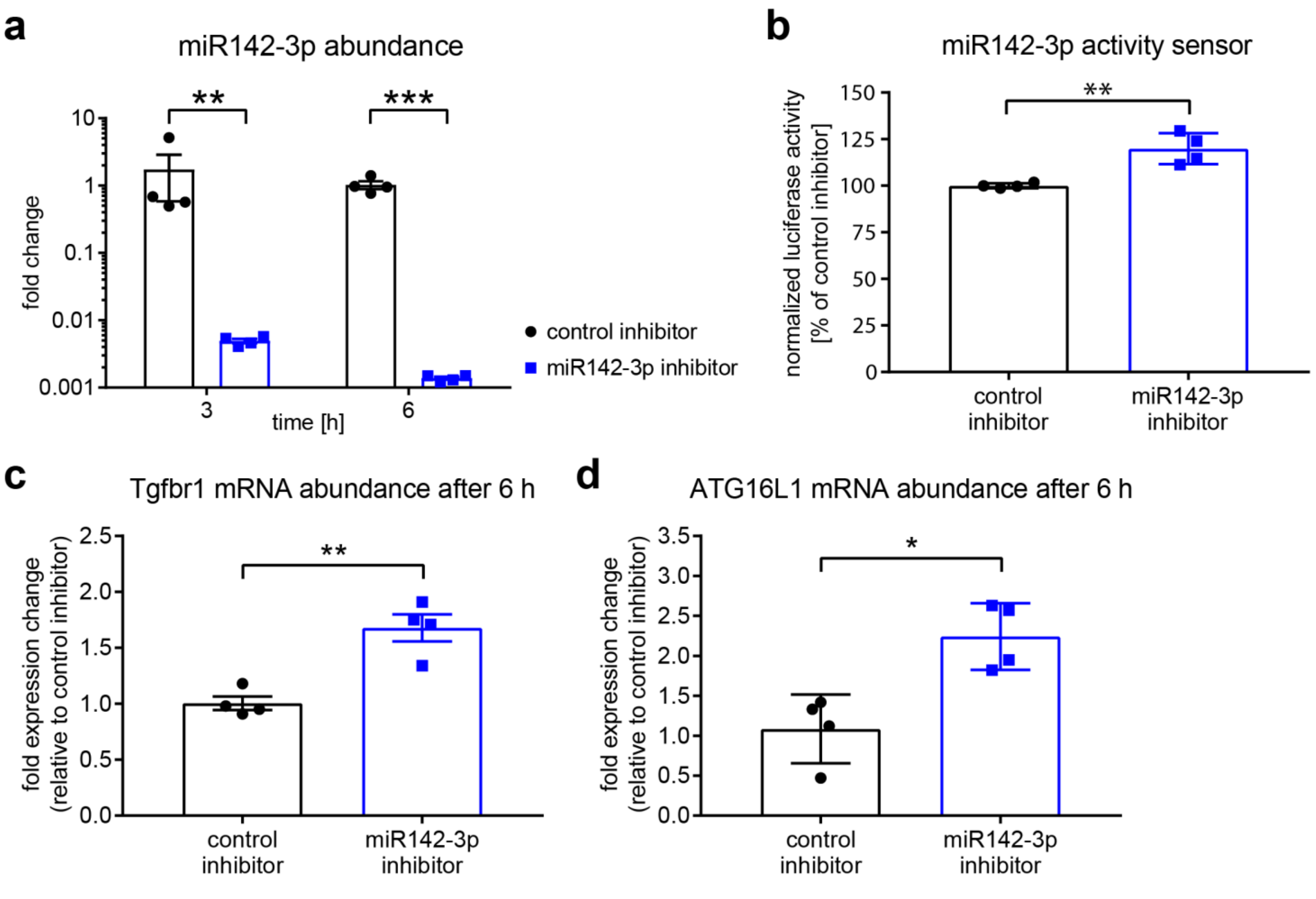

Supplementary Figure 6. miR142-3p inhibitor efficiently blocks its target miRNA. (a) Effect of the miR142-3p inhibitor on levels of miR142-3p upon TCR stimulation of naive $CD4^+$ T cells isolated from lymph nodes of BALB/c mice as revealed by qPCR normalized to 5s rRNA. n = 4. **(b)** Normalized luciferase activity of Jurkat T cells co-transfected with a miR142-3p inhibitor and a miR142-3p activity sensor plasmid containing the miR142-3p target sequences in the 3'-UTR. n = 4. **(c)** Levels of Tgfbr1 mRNA, an established target of miR142-3p, after 6 hours of TCR stimulation of $CD4^+$ T cells isolated from lymph nodes of BALB/c mice, in presence of a miR142-3p inhibitor and a control inhibitor respectively as revealed by qPCR normalized to Histone mRNA. n = 4. **(d)** Levels of ATG16L1 mRNA, an established target of miR142-3p, after 6 hours of TCR stimulation of $CD4^+$ T cells isolated from lymph nodes of BALB/c mice, in presence of a miR142-3p inhibitor and a control inhibitor respectively as revealed by qPCR normalized to Histone mRNA. n = 4. One data point represents one subject. Experiments were performed in three technical replicates per subject. Data are presented as box-and-whisker plots with min and max values or as means ± SEM. (a) Ordinary one-way ANOVA, Tukey's multiple comparisons test. (b, c and d) Student's t-test. *$P < 0.05$, **$P < 0.01$, ***$P < 0.001$. Source data are provided as a Source Data file.

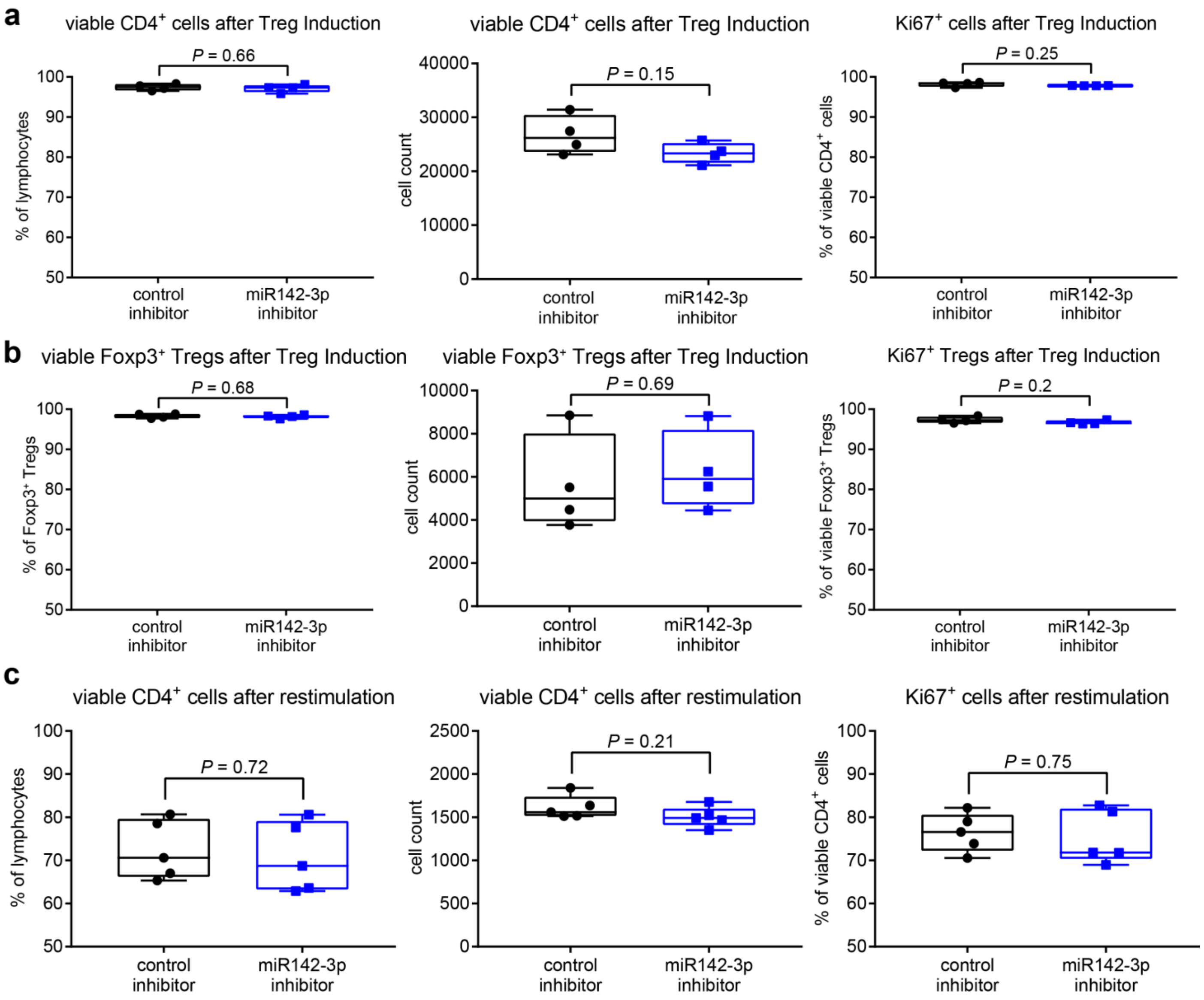

Supplementary Figure 7. miR142-3p inhibition does not affect cell viability or proliferation during Treg induction or restimulation *in vitro*. (a) Cell viability (% and cell count) and Ki67$^+$ cells of CD4$^+$ T cells after Treg induction using subimmunogenic stimulation of naive CD4$^+$ T cells isolated from lymph nodes of BALB/c mice as revealed by flow cytometry. n = 4. **(b)** Cell viability (% and cell count) and Ki67$^+$ cells of Foxp3$^+$ Tregs after induction in vitro using subimmunogenic stimulation of naive CD4$^+$ T cells isolated from lymph nodes of BALB/c mice as revealed by flow cytometry. n = 4. **(c)** Cell viability (% and cell count) and Ki67$^+$ cells of CD4$^+$ T cells after restimulation of *in vitro* induced Tregs for 30 hours as revealed by flow cytometry. n = 5. One data point represents one subject. Experiments were performed in three technical replicates per subject. Data are presented as box-and-whisker plots with min and max values. Student's t-test. Source data are provided as a Source Data file.

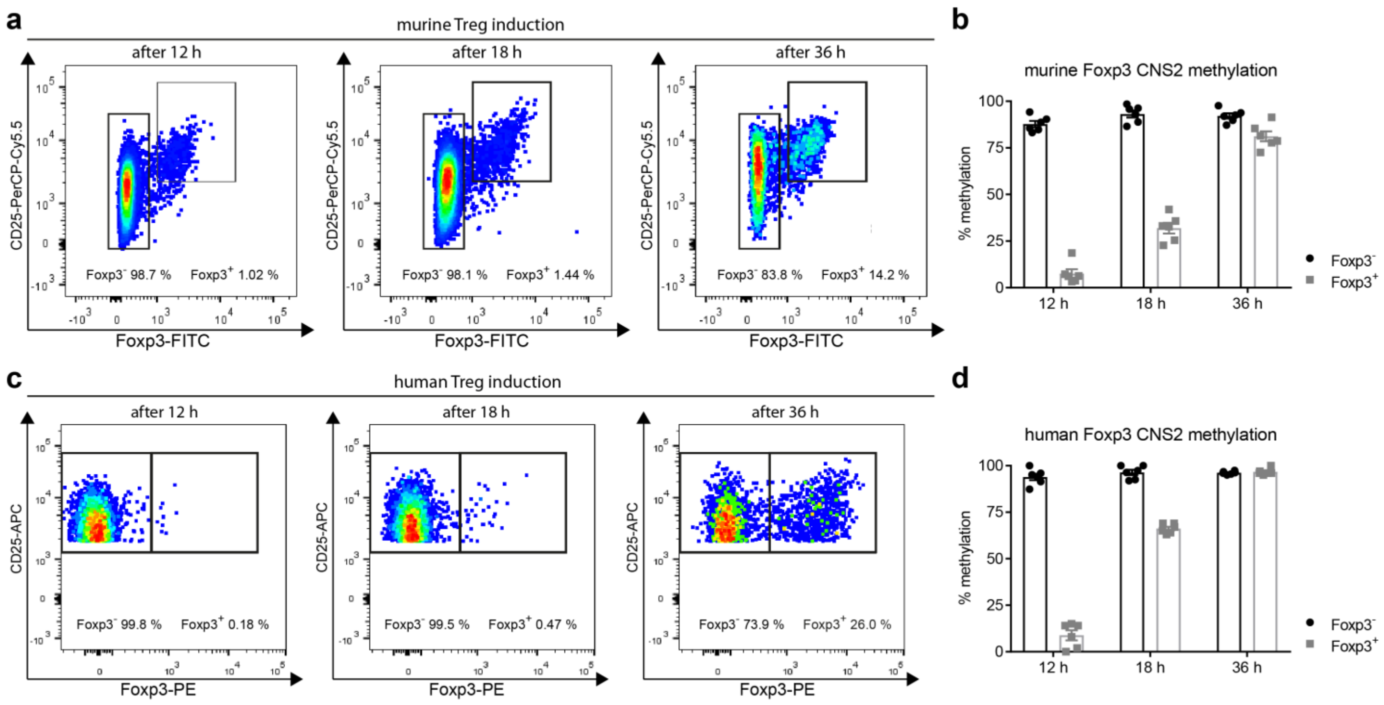

Supplementary Figure 8. Early T cell activation induces rapid demethylation of the Foxp3 CNS2 only in Foxp3$^+$ cells. (a) Representative flow cytometry plot indicating CD4$^+$CD25$^+$Foxp3$^+$ Tregs and CD4$^+$Foxp3$^-$ T cells after 12, 18 and 36 hours of subimmunogenic stimulation of BALB/c naive CD4$^+$ T cells. **(b)** Methylation of four CpG sites in the murine Foxp3 CNS2 of CD4$^+$CD25$^+$Foxp3$^+$ Tregs and CD4$^+$Foxp3$^-$ T cells after 12, 18 and 36 hours of subimmunogenic TCR stimulation as revealed by pyrosequencing. *n* = 6. **(c)** Representative flow cytometry plot indicating CD4$^+$CD127$^-$CD25$^+$Foxp3$^+$ Tregs and CD4$^+$CD127$^-$CD25$^+$Foxp3$^-$ T cells after 12, 18 and 36 hours of TCR stimulation of human naive CD4$^+$ T cells. **(d)** Methylation of eight CpG sites in the human Foxp3 CNS of CD4$^+$CD127$^-$CD25$^+$Foxp3$^+$ Tregs and CD4$^+$CD127$^-$CD25$^+$Foxp3$^-$ T cells after 12, 18 and 36 hours of subimmunogenic TCR stimulation as revealed by pyrosequencing. *n* = 6. Source data are provided as a Source Data file.

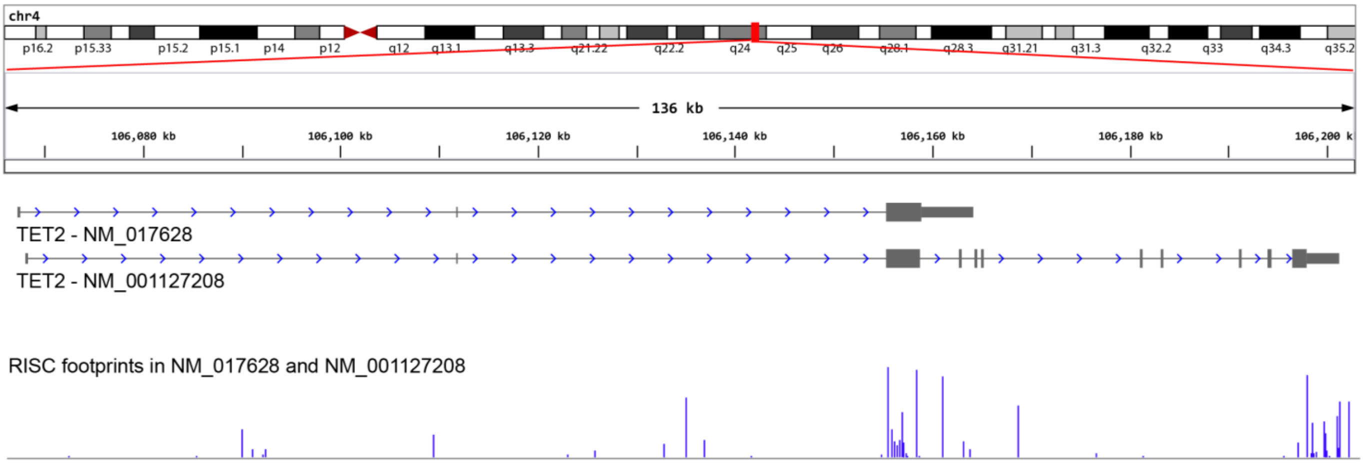

Supplementary Figure 9. HITS-CLIP analysis of human CD4^{+} T cells. From top to bottom: Location of the human TET2 gene on chromosome 4. The human TET2 transcripts NM_017628 and NM_001127208 and RISC footprints (purple bars) on these transcripts.

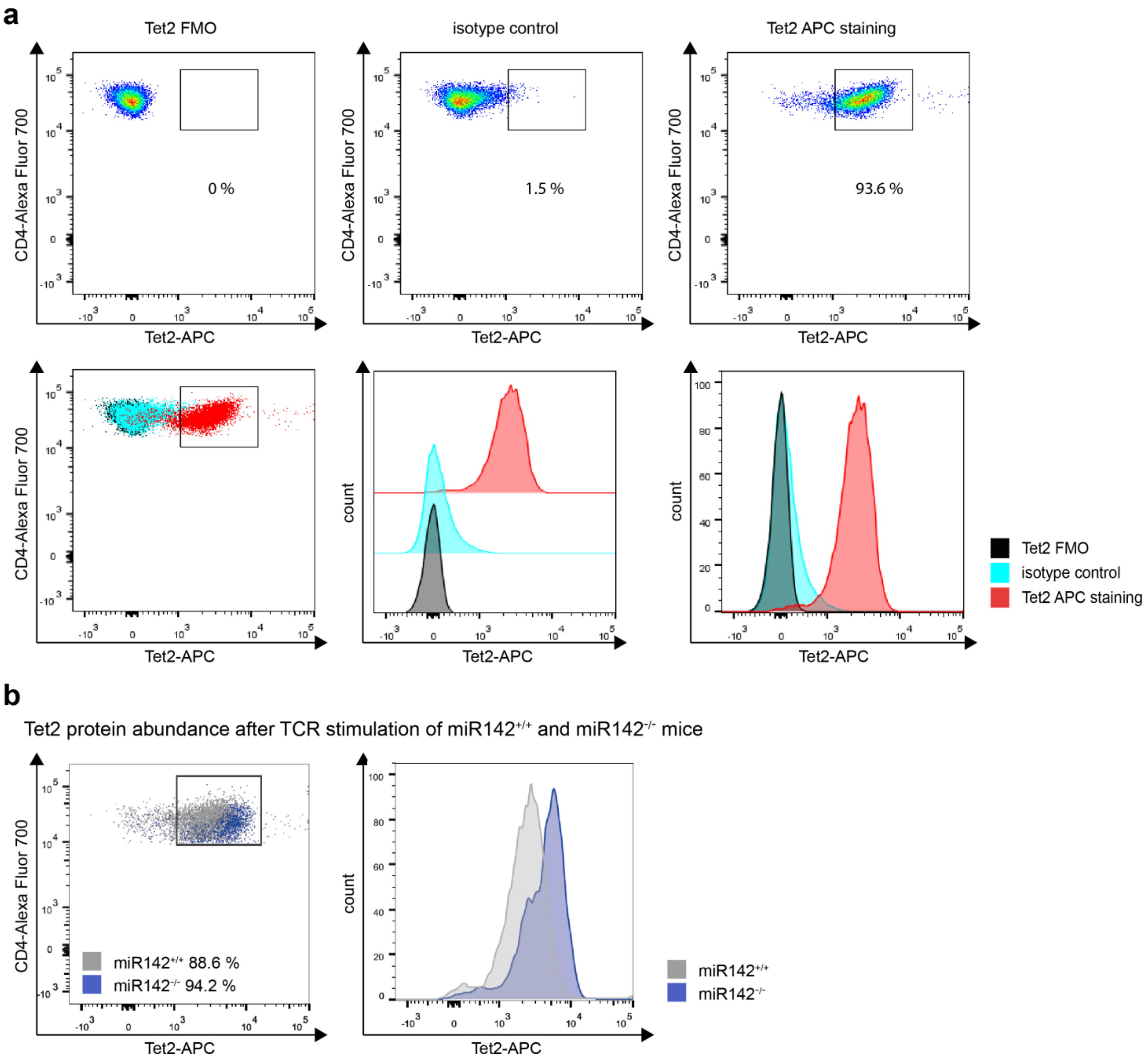

Supplementary Figure 10. Tet2 flow cytometry staining of murine CD4^{+} T cells. (a) Upper panel: pseudocolor dot plots showing FMO, isotype control and Tet2-APC staining. Lower panel: overlay of FMO, isotype control and Tet2-APC dot plots (left) and histograms showing FMO, isotype control and Tet2-APC staining (middle and right). **(b)** Dot plots (left) and histograms (right) of a representative Tet2 flow cytometry staining of CD4^{+} T cells isolated from lymph nodes of miR142$^{+/+}$ and miR142$^{-/-}$ mice.

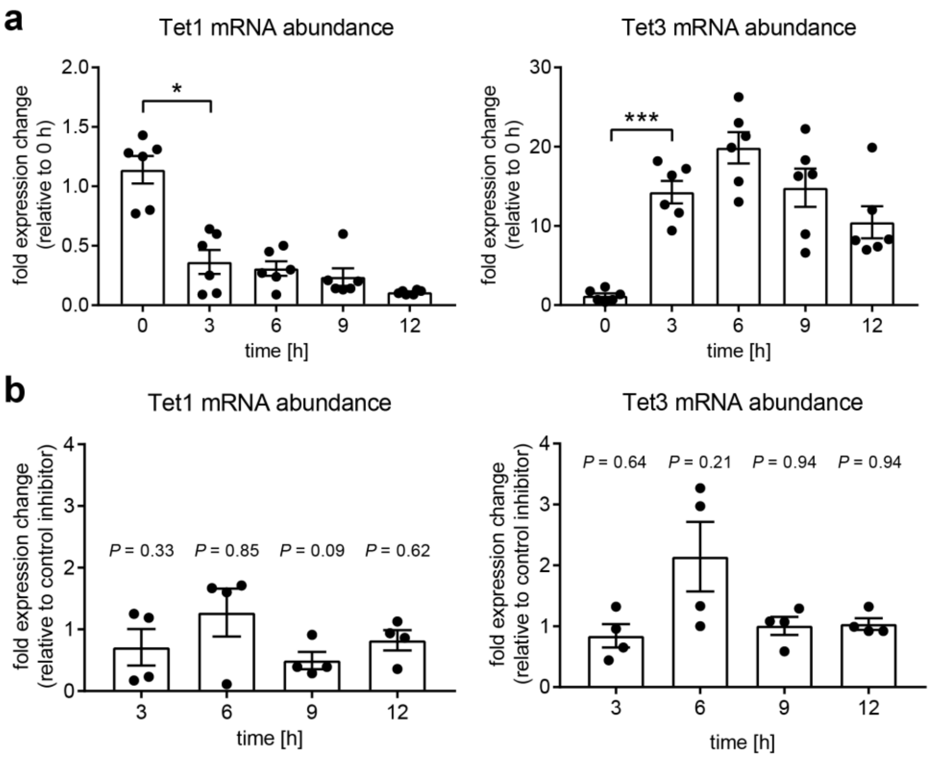

Supplementary Figure 11. Tet1 and Tet3 expression upon TCR stimulation and miR142-3p inhibition. (a) Levels of Tet1 and Tet3 mRNA at various time points (0 – 12 hours) of TCR stimulation of $CD4^+$ T cells isolated from lymph nodes of BALB/c mice as revealed by qPCR normalized to Histone mRNA. $n = 6$. **(b)** Levels of Tet1 and Tet3 mRNA after 3, 6, 9 and 12 hours of TCR stimulation of $CD4^+$ T cells isolated from lymph nodes of BALB/c mice, in presence of a miR142-3p inhibitor and a control inhibitor respectively as revealed by qPCR normalized to Histone mRNA. $n = 4$. One data point represents one subject. Experiments were performed in three technical replicates per subject. Data are presented as means ± SEM. Ordinary one-way ANOVA, Tukey's multiple comparisons test. $*P < 0.05$, $***P < 0.001$. Source data are provided as a Source Data file.

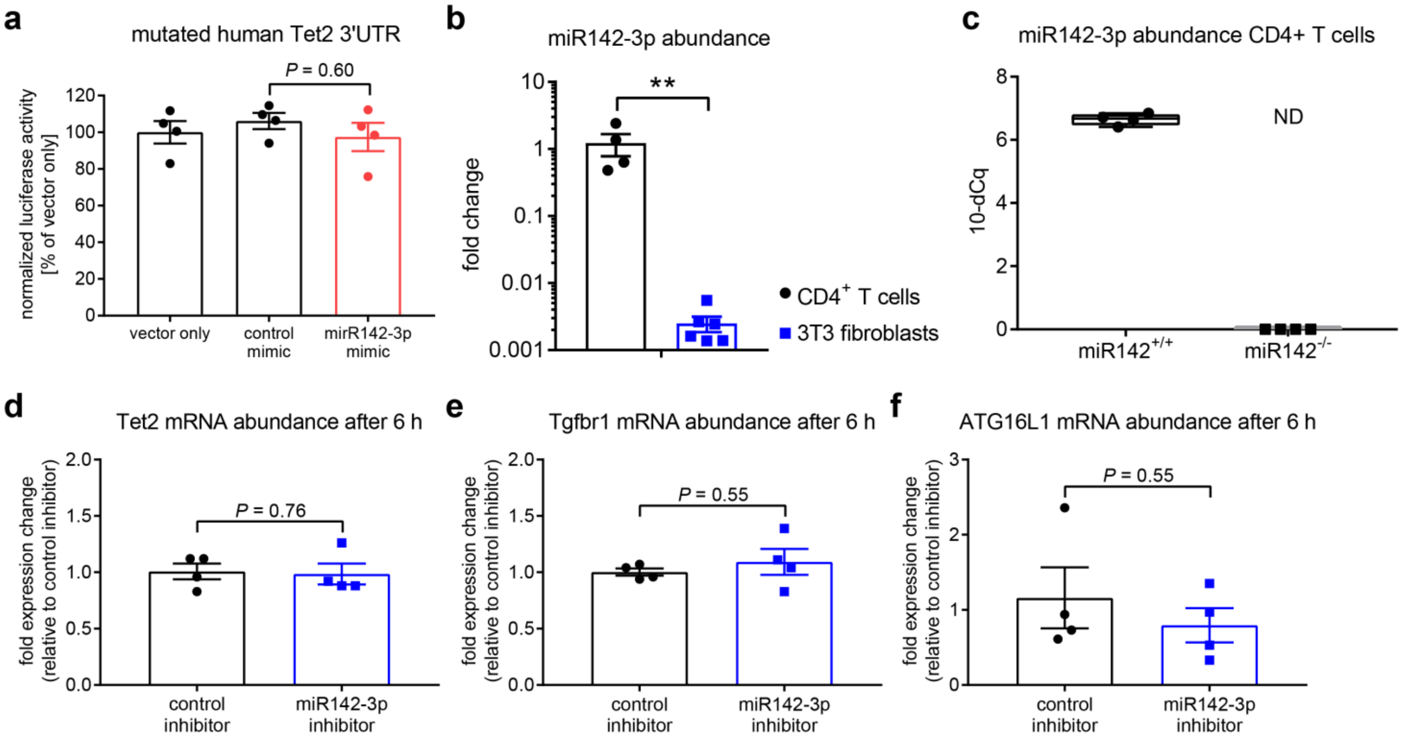

Supplementary Figure 12. miR142 knockout models and effect of miR142-3p inhibitor in miR142$^{-/-}$ CD4^{+} T cells *in vitro*. **(a)** Normalized luciferase activity of HEK-293 cells co-transfected with a miR142-3p mimic and a TET2 3′UTR reporter construct, with a mutation in the predicted miR142-3p binding sites. n = 4. **(b)** Levels of miR142-3p in CD4^{+} T cells isolated from lymph nodes of BALB/c mice and 3T3 fibroblasts as revealed by qPCR normalized to 5s rRNA. n = 4. **(c)** Levels of miR142-3p in CD4^{+} T cells isolated from lymph nodes of miR142 knockout (miR142$^{-/-}$) and control (miR142$^{+/+}$) mice as revealed by qPCR normalized to 5s rRNA. n = 4. ND = not detectable. **(d)** Levels of Tet2 mRNA after 6 hours of TCR stimulation of CD4^{+} T cells isolated from lymph nodes of miR142$^{-/-}$ mice, in presence of a miR142-3p inhibitor and a control inhibitor respectively as revealed by qPCR normalized to Histone mRNA. n = 4. **(e)** Levels of Tgfbr1 mRNA after 6 hours of TCR stimulation of CD4^{+} T cells isolated from lymph nodes of miR142$^{-/-}$ mice, in presence of a miR142-3p inhibitor and a control inhibitor respectively as revealed by qPCR normalized to Histone mRNA. n = 4. **(f)** Levels of ATG16L1 mRNA after 6 hours of TCR stimulation of CD4^{+} T cells isolated from lymph nodes of miR142$^{-/-}$ mice, in presence of a miR142-3p inhibitor and a control inhibitor respectively as revealed by qPCR normalized to Histone mRNA. n = 4. One data point represents one subject. Experiments were performed in three technical replicates per subject. Data are presented as box-and-whisker plots with min and max values or as means ± SEM. Student's t-test. $^{**}P < 0.01$. Source data are provided as a Source Data file.

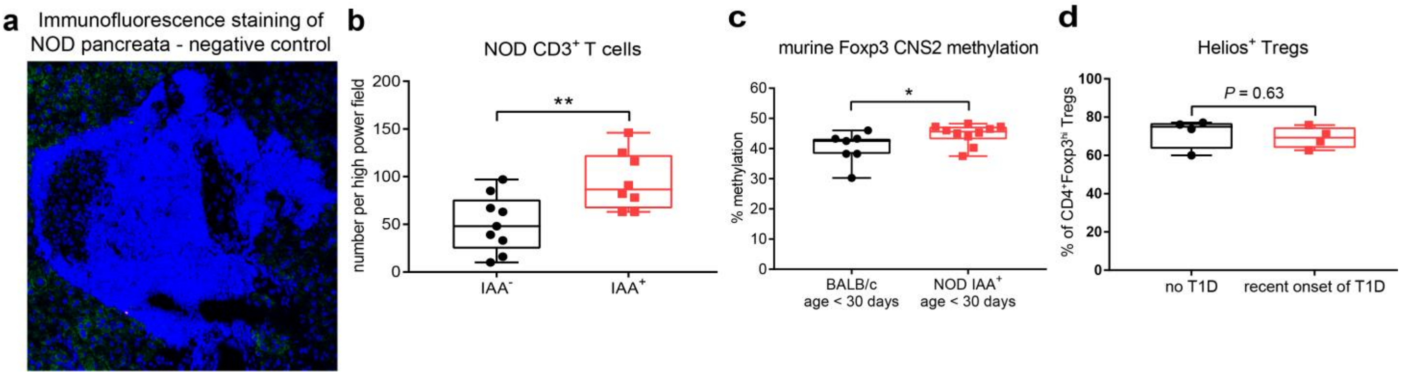

Supplementary Figure 13. Tet2 abundance and Foxp3 CNS2 methylation are changed upon onset of islet autoimmunity. (a) Negative control for immunofluorescence staining of pancreas cryosections as shown in Fig. 7b (DAPI = blue). **(b)** $CD3^+$ T cells per high power field in pancreas cryosections of NOD mice with and without islet autoimmunity as shown in Fig. 7b. $n \geq 8$. **(c)** Methylation of the Foxp3 CNS2 (mean of all sites) in Tregs isolated from lymph nodes of young female BALB/c mice or IAA^+ NOD mice, age < 30 days as revealed by pyrosequencing. $n \geq 7$. **(d)** $Helios^+$ Tregs (% of $CD4^+Foxp3^{hi}$ Tregs) isolated from peripheral blood of human subjects with recent onset of T1D and healthy controls as revealed by flow cytometry. $n = 4$. One data point represents one subject / one high power field. Experiments were performed in three technical replicates per subject. Data are presented as box-and-whisker plots with min and max values. Student’s t-test. $*P < 0.05$, $**P < 0.01$. Source data are provided as a Source Data file.

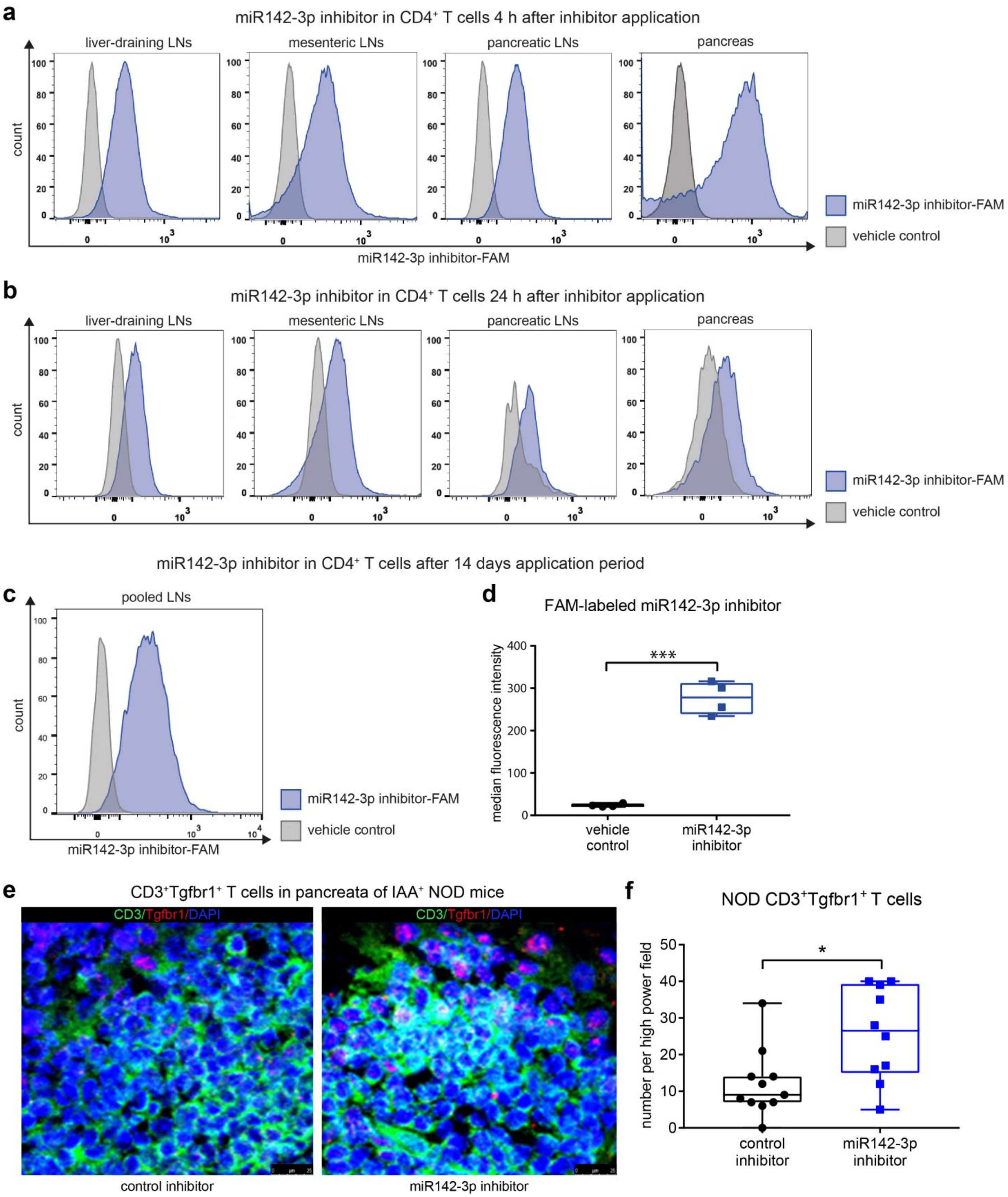

Supplementary Figure 14. miR142-3p inhibitor accumulates in CD4$^+$ T cells *in vivo*. (a) Representative flow cytometry histograms showing the accumulation of the FAM-labelled miR142-3p inhibitor in CD4$^+$ T cells isolated from relevant lymph nodes and the pancreas 4 hours after inhibitor application. **(b)** Representative flow cytometry histograms showing the accumulation of the FAM-labelled miR142-3p inhibitor in CD4$^+$ T cells isolated from relevant lymph nodes and the pancreas 24 hours after inhibitor application. **(c)** Representative flow cytometry histograms showing the accumulation of the FAM-labelled miR142-3p inhibitor in CD4$^+$ T cells isolated from lymph nodes of mice treated with a miR142-3p inhibitor

or control inhibitor for 14 days with 10 mg/kg ip every other day. **(d)** miR142-3p inhibitor abundance (median fluorescence intensity) in $CD4^+$ T cells isolated from lymph nodes of mice treated as described in (c) and revealed by flow cytometry. $n = 4$. **(e)** Immunofluorescent staining for CD3 (green), Tgfbr1 (red) and DAPI (blue) in pancreas cryosections of IAA^+ NOD mice after treatment with a miR142-3p inhibitor or control inhibitor as described in (c). Scale bar: 25 µm **(f)** Quantification of $CD3^+Tgfbr1^+$ T cells per high power field in samples from (e). $n \geq 10$. One data point represents one mouse (d), one high power field (f). Data are presented as box-and-whisker plots with min and max values. Student's t-test. $*P < 0.05$, $***P < 0.001$. Source data are provided as a Source Data file.

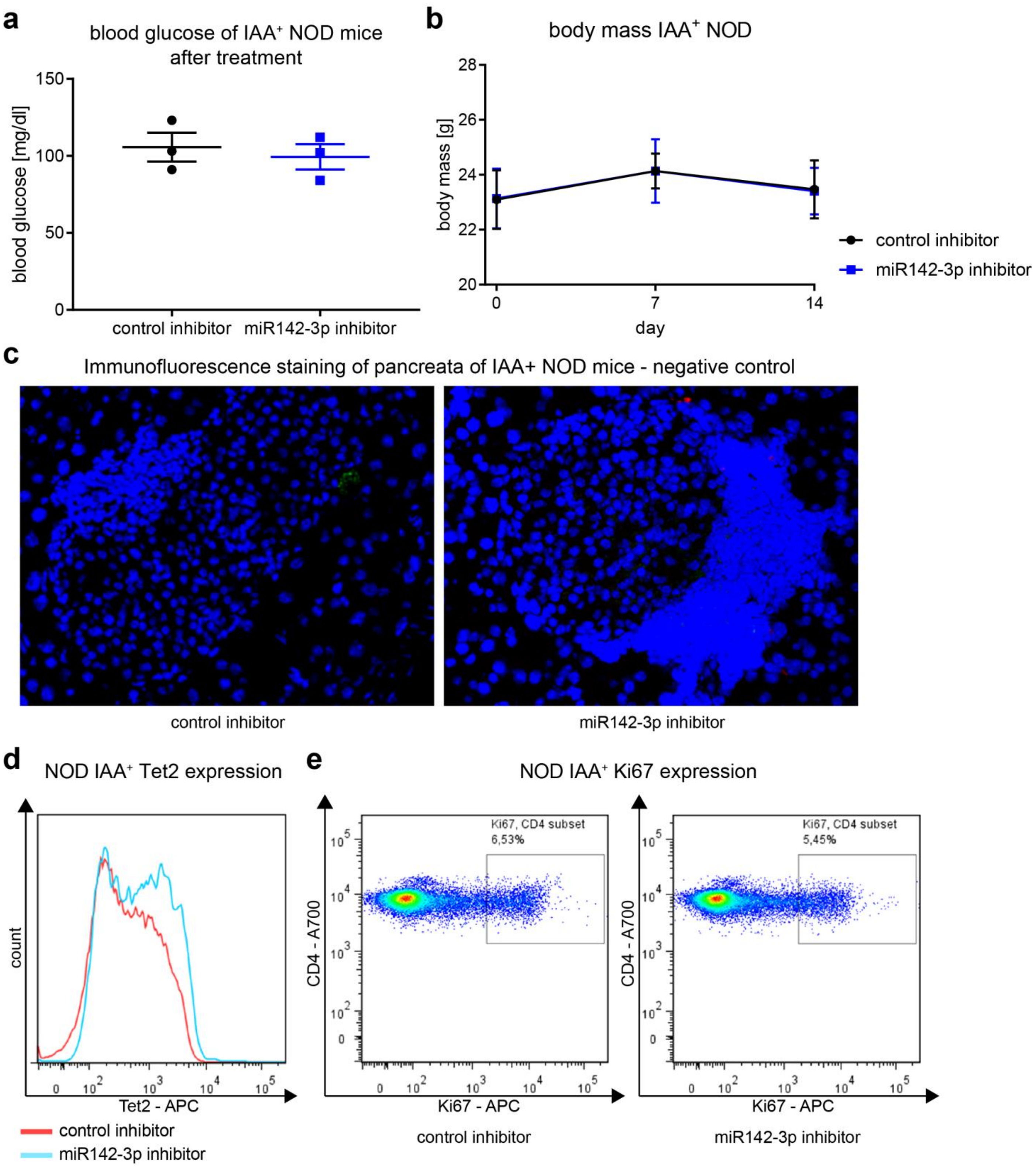

Supplementary Figure 15. Effects of miR142-3p inhibition *in vivo*. (a) Blood glucose levels in IAA$^+$ NOD mice treated with a miR142-3p inhibitor or control inhibitor for 14 days with 10 mg/kg ip every other day. n = 3. **(b)** Body mass development in IAA$^+$ NOD mice treated as described in (a). n = 3. **(c)** Negative control for immunofluorescence staining of pancreas cryosections of IAA$^+$ NOD mice treated as described in (a) and as shown in Fig. 8d (DAPI = blue). **(d)** Representative flow cytometry histogram of Tet2 staining in CD4$^+$ T cells isolated from peripheral blood of IAA$^+$ NOD mice treated as described in (a). **(e)** Representative flow cytometry plots indicating CD4$^+$Ki67$^+$ T cells in peripheral blood of IAA$^+$ NOD mice treated as described in (a). One data point represents one mouse (a), mean of three mice (b). Data are

presented as means ± SEM. (b) Ordinary one-way ANOVA, Tukey's multiple comparisons test. (a) Student’s t-test. *P < 0.05. Source data are provided as a Source Data file.

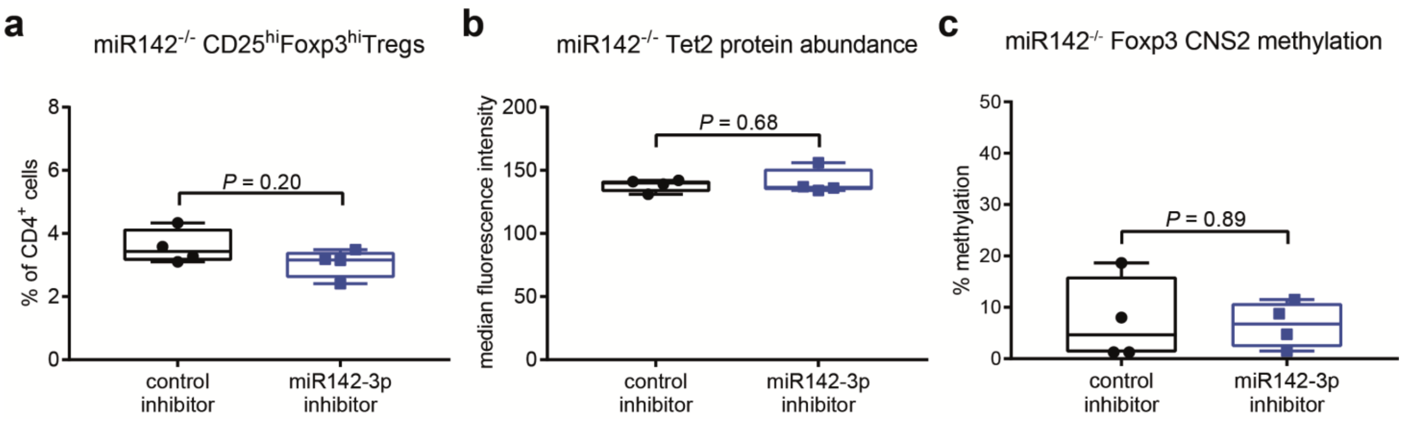

Supplementary Figure 16. miR142-3p inhibitor has no effect in miR142$^{-/-}$ mice *in vivo*. (a) Ex *vivo* CD25^{hi}Foxp3^{+} Tregs isolated from lymph nodes of miR142$^{-/-}$ mice treated with a miR142-3p inhibitor or control inhibitor for 14 days with 10 mg/kg ip every other day as revealed by flow cytometry. n = 4. **(b)** *Ex vivo* Tet2 protein abundance (median fluorescence intensity) in CD4^{+} T cells isolated from lymph nodes of miR142$^{-/-}$ mice treated with a miR142-3p inhibitor or control inhibitor as described in (a) and revealed by flow cytometry. n = 4. **(c)** Methylation of the Foxp3 CNS2 (mean of all sites) in Tregs isolated from lymph nodes of miR142$^{-/-}$ mice treated with a miR142-3p inhibitor or control inhibitor as described in (a) and revealed by pyrosequencing. n = 4. One data point represents one mouse. Data are presented as box-and-whisker plots with min and max values. Student's t-test. Source data are provided as a Source Data file.

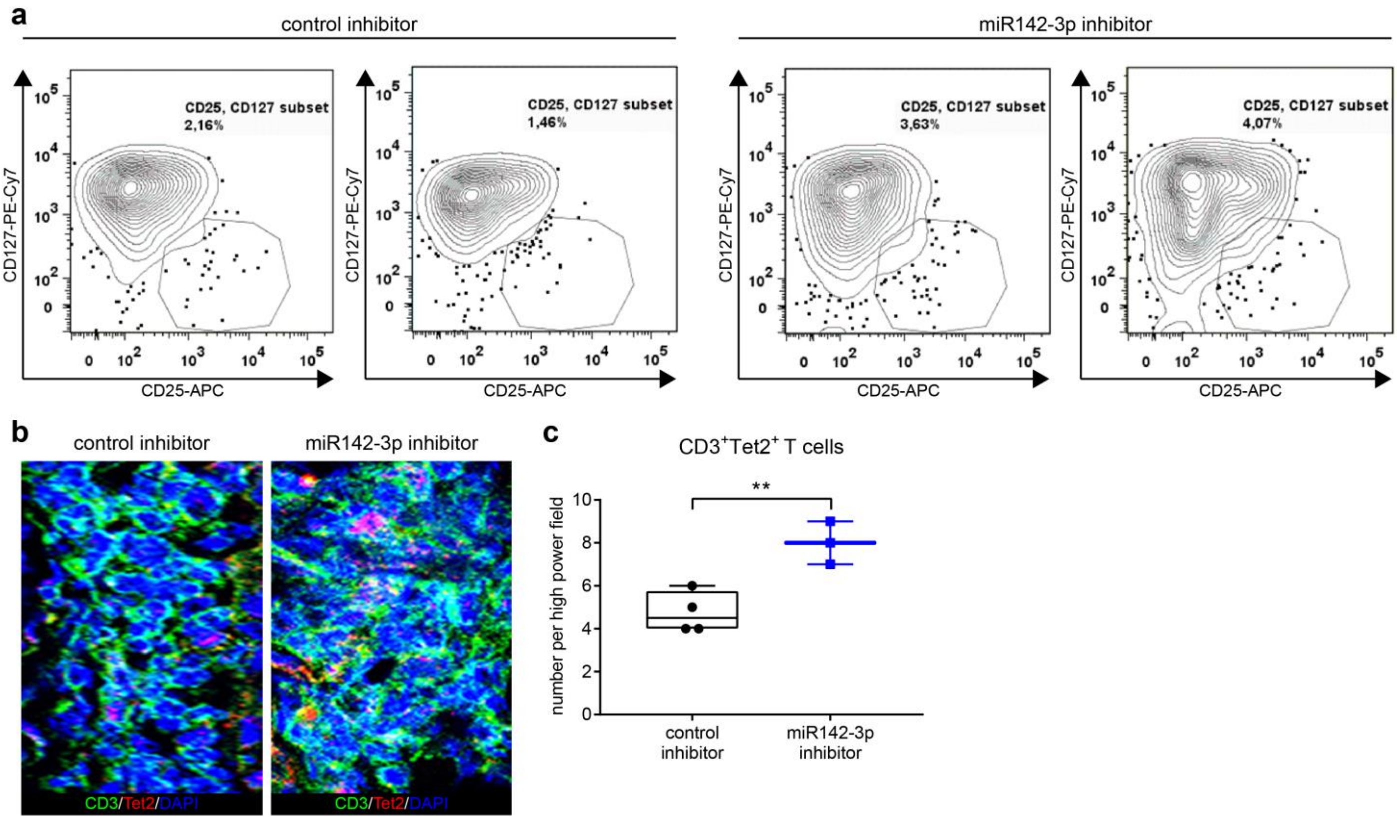

Supplementary Figure 17. Effects of miR142-3p inhibition in humanized NSG mice. (a) Representative set of flow cytometry plots indicating CD25^{hi}CD127^{lo} Tregs in lymph nodes and spleen of humanized NSG mice treated with a miR142-3p inhibitor or control inhibitor for 14 days with 10 mg/kg ip every other day. **(b)** Immunofluorescent staining for CD3 (green), Tet2 (red) and DAPI (blue) in pancreas cryosections of humanized NSG mice treated with a miR142-3p inhibitor or control inhibitor as described in (a). **(c)** Quantification of CD3^{+}Tet2^{+} T cells per high power field in samples from (b). $n \geq 3$. One data point represents one high power field. Data are presented as box-and-whisker plots with min and max values Student's t-test. $**P < 0.01$. Source data are provided as a Source Data file.

Supplementary Table 1: Oligonucleotide sequences

Primer sequences qPCR

gene	forward	reverse	source
Histone	ACTGGCTACAAAAGCCG	ACTTGCCTCCTGCAAAGCAC	Sigma Aldrich
Tet2 hs	TTCGCAGAAGCAGCAGTGAAGAG	AGCCAGAGACAGCGGGATTCCTT	Sigma Aldrich
Tet1 mm	ATGAGCGGCACCCTGAAGCG	GCACCGAGCCGTGAATGGGT	Sigma Aldrich
Tet2 mm	CCTTGCATTGGAGGGGTGGCT	GTGGGGTGATTCCGGTCGGG	Sigma Aldrich
Tet3 mm	CACGGCTTCGAGGCAAGCCA	CCCCGGTTCCCATCCCCCAT	Sigma Aldrich
Tgfbr1	GCAGCTCCTCATCGTGTTGG	GCAGAAACACTGTAATGCCTTCG	Sigma Aldrich
ATG16L1	CCGAATCTCCCCTTTTGGGA	CATGCGCATCGAAGACATACG	Sigma Aldrich
5s	N/A	N/A	Exiqon (miRCURY LNA primer set)
miR142-3p hs	N/A	N/A	Exiqon (miRCURY LNA primer set)
miR142-3p mm	N/A	N/A	Exiqon (miRCURY LNA primer set)

Primer sequences methylation analysis

site	forward	reverse	pyrosequencing	source
Foxp3 CNS2 hs	AAGTTGAATGGGGGATGTTTTTGGGATATAGATTATG	CTACCACATCCACCAACACCCATATCACC	TAGTTTTAGATTTGTTTAGATTTT	Sigma Aldrich
Foxp3 CNS2 mm	TTGGGTTTTGTTGTTATAATTTGAATTTGG	ACCTACCTAATACTCACCAAACATC	AATTTGAATTTGGTTAGATTTTT	Sigma Aldrich

Primer sequences for site directed mutagenesis of TET2 3’UTR

site	forward (mutation in bold type)	reverse (mutation in bold type)	source
mut_site1	GTCATATACCTCAAACATAGTTTGGCAATAGG	CCTATTGCCAAACTATGTTTGAGGTATATGAC	Sigma Aldrich
mut_site2	CTTTTGCAGTTTGAACATAAGATAACTTCTGTG	CACAGAAGTTATCTTATGTTCAAACTGCAAAAG	Sigma Aldrich

Sequences miRNA inhibitors and mimics

inhibitor/mimic	sequence	source
miR142-3p inhibitor hs/mm	CCATAAAGTAGGAAACACT	Exiqon miRCURY LNA™ microRNA inhibitor
miR142-3p mimic hs/mm	TGTAGTGTTTCCTACTTTATGGA	Exiqon miRCURY LNA™ microRNA mimic
control inhibitor hs/mm	TAACACGTCTATACGCCCA	Exiqon miRCURY LNA™ microRNA inhibitor
control mimic hs/mm	GATGGCATTCGATCAGTTCTA	Exiqon miRCURY LNA™ microRNA mimic

Sequences siRNAs

siRNA	sequence	source
Tet2 mm	N/A	Ambion (Silencer Select Pre-Designed siRNA)
Tet2 hs	N/A	Ambion (Silencer Select Pre-Designed siRNA)
Negative control	N/A	Ambion (Silencer Select Negative control no.1)
