# Supplementary material for: miRNA142-3p targets Tet2 and impairs Treg differentiation and stability in models of type 1 diabetes
Source: Nat Commun. 2019 Dec 13;10:5697. doi: 10.1038/s41467-019-13587-3 (PMC6910913; doi:10.1038/s41467-019-13587-3)
Supplement: Supplementary file 3 — Reporting Summary [file 41467_2019_13587_MOESM3_ESM.pdf]

nature research

Corresponding author(s): Carolin Daniel

Last updated by author(s): Nov 4, 2019

Reporting Summary

Nature Research wishes to improve the reproducibility of the work that we publish. This form provides structure for consistency and transparency in reporting. For further information on Nature Research policies, see Authors & Referees and the Editorial Policy Checklist.

Statistics

For all statistical analyses, confirm that the following items are present in the figure legend, table legend, main text, or Methods section.

n/a	Confirmed	
☐	☒	The exact sample size (*n*) for each experimental group/condition, given as a discrete number and unit of measurement
☐	☒	A statement on whether measurements were taken from distinct samples or whether the same sample was measured repeatedly
☐	☒	The statistical test(s) used AND whether they are one- or two-sided<br>*Only common tests should be described solely by name; describe more complex techniques in the Methods section.*
☐	☒	A description of all covariates tested
☐	☒	A description of any assumptions or corrections, such as tests of normality and adjustment for multiple comparisons
☐	☒	A full description of the statistical parameters including central tendency (e.g. means) or other basic estimates (e.g. regression coefficient) AND variation (e.g. standard deviation) or associated estimates of uncertainty (e.g. confidence intervals)
☐	☒	For null hypothesis testing, the test statistic (e.g. *F*, *t*, *r*) with confidence intervals, effect sizes, degrees of freedom and *P* value noted<br>*Give P values as exact values whenever suitable.*
☒	☐	For Bayesian analysis, information on the choice of priors and Markov chain Monte Carlo settings
☒	☐	For hierarchical and complex designs, identification of the appropriate level for tests and full reporting of outcomes
☒	☐	Estimates of effect sizes (e.g. Cohen's *d*, Pearson's *r*), indicating how they were calculated

Our web collection on statistics for biologists contains articles on many of the points above.

Software and code

Policy information about availability of computer code

Data collection	FACSDiva software (version 6.1.3)
Data analysis	FlowJo software (version 7.6.1)<br>GraphPad Prism (version 6.0.1)<br>miRanda (v3.3a)<br>BTrim<br>bowtie<br>bowtie2<br>HTSeq-count<br>mirBase (release 20)<br>DESeq2

For manuscripts utilizing custom algorithms or software that are central to the research but not yet described in published literature, software must be made available to editors/reviewers. We strongly encourage code deposition in a community repository (e.g. GitHub). See the Nature Research guidelines for submitting code & software for further information.

Data

Policy information about availability of data

All manuscripts must include a data availability statement. This statement should provide the following information, where applicable:

- Accession codes, unique identifiers, or web links for publicly available datasets
- A list of figures that have associated raw data
- A description of any restrictions on data availability

The authors declare that all data supporting the findings of the study are available within the article and its Supplementary Material or from the corresponding author upon reasonable request. The source data underlying Figures 1g and h, 2a and c-f, 3a and c-e, 4b, d and f, 5a-j, 6a, c and e-g, 7b, c, e g and h and

Supplementary Figures 3, 6a-d, 7a-c, 8b and d, 11a and b, 12a-f, 13b-d, 14d and f, 15a and b, 16a-c and 17c are provided as a Source Data file. All HITS-CLIP library sequencing data have been deposited into NCBI GEO under accession number GSE124264.

Field-specific reporting

Please select the one below that is the best fit for your research. If you are not sure, read the appropriate sections before making your selection.

☒ Life sciences ☐ Behavioural & social sciences ☐ Ecological, evolutionary & environmental sciences

For a reference copy of the document with all sections, see nature.com/documents/nr-reporting-summary-flat.pdf

Life sciences study design

All studies must disclose on these points even when the disclosure is negative.

Sample size	No sample-size calculation was performed. Sample size was determined based on similar studies in previous literature and based on the magnitude and consistency of differences between groups.
Data exclusions	Data were only excluded for failed experiments, e.g. suboptimal Treg induction in all groups because of technical issues.
Replication	Replication experiments were successful.
Randomization	No randomization of mice. Mice analyzed were litter mates and sex-matched whenever possible.
Blinding	Investigators were blinded for the quantification of immunofluorescence microscopy experiments.

Reporting for specific materials, systems and methods

We require information from authors about some types of materials, experimental systems and methods used in many studies. Here, indicate whether each material, system or method listed is relevant to your study. If you are not sure if a list item applies to your research, read the appropriate section before selecting a response.

Materials & experimental systems

n/a	Involved in the study	
☐	☒	Antibodies
☐	☒	Eukaryotic cell lines
☒	☐	Palaeontology
☐	☒	Animals and other organisms
☐	☒	Human research participants
☒	☐	Clinical data

Methods

n/a	Involved in the study	
☒	☐	ChIP-seq
☐	☒	Flow cytometry
☒	☐	MRI-based neuroimaging

Antibodies

Antibodies used

FACS antibodies:
anti-human CD25 APC, BD Biosciences, Cat# 340907, clone: 2A3; anti-human CD45RO APC-H7, BD Biosciences, Cat# 561137, clone: UCHL1; anti-human CD4 V500, BD Biosciences, Cat# 560768, clone: RPA-T4; anti-human HLA-DR PerCP-Cy5.5, BD Biosciences, Cat# 560652, clone: L243; anti-human CD45RA FITC, Biolegend, Cat# 304106, clone: HI100; anti-human CD3 PerCP-Cy5.5, Biolegend, Cat# 300328, clone: HIT3a; anti-human CD3 AlexaFluor700, Biolegend, Cat# 300323, clone: HIT3a; anti-human CD127 PE-Cy7, Biolegend, Cat# 351320, clone: A019D5; anti-human FOXP3 PE, eBioscience, Cat# 12-4777-42, clone: 236A/E7; anti-mouse CD4 Biotin, BD Biosciences, Cat# 553728, clone: GK1.5; anti-mouse CD4 AlexaFluor700, eBioscience, Cat# 56-0042-82, clone: RM4-5; anti-mouse CD25 PerCP-Cy5.5, Biolegend, Cat# 102030, clone: PC61; anti-mouse CD44 PE, Biolegend, Cat# 103008, clone: IM7; anti-mouse Ki67 APC, Biolegend, Cat# 652406, clone: 16A8; anti-mouse CD62L APC, eBioscience, Cat# 17-0621-82, clone: MEL-14; anti-mouse Foxp3 FITC, eBioscience, Cat# 11-5773-82, clone: FJK-16s; anti-mouse Tet2, Abiocode, Cat# AC-M1086-4a, clone: 10F1; donkey-anti-mouse IgG APC, eBioscience, Cat# 17-4012-82, polyclonal
Immunofluorescence antibodies:
insulin rabbit-anti-mouse, Cell Signaling; donkey-anti-rabbit AlexaFluor647, Dianova; CD3 arm. hamster-anti-mouse, BD Biosciences; goat-anti-arm.hamster Dylight488, Dianova; Tet2, ABclonal, clone: A5682; horse-anti-rabbit Biotin, Vector; Streptavidin Dylight549, Dianova; rat-anti-mouse Foxp3, eBioscience, clone: FJK-16s; goat anti-rabbit Biotin, BD Biosciences; rat-anti-mouse/human Tgfbr1, R&D, clone 141231
coating antibodies:
anti-human CD3, Biolegend, Cat# 300432, clone: UCHT1; anti-human CD28, Biolegend, Cat# 302923, clone: CD28.2; anti-mouse CD3, BD Pharmingen, Cat# 553057, clone: 145-2C11; anti-mouse CD28, BD Pharmingen, Cat# 553294, clone: 37.51

Validation	All antibodies are commercially available and were previously validated for species and application (manufacturer's website) and/or commonly used in publications.

Eukaryotic cell lines

Policy information about cell lines

Cell line source(s)	3T3 Fibroblasts and HEK293 cells were obtained from IDO, Helmholtz Zentrum München
Authentication	Cell lines were authenticated by the providers.
Mycoplasma contamination	Cell lines were tested negative for mycoplasma contamination.
Commonly misidentified lines (See ICLAC register)	N/A

Animals and other organisms

Policy information about studies involving animals; ARRIVE guidelines recommended for reporting animal research

Laboratory animals	NOD/ShiLtJ mice and NOD.Cg-Prkdcscid H2-Ab1tm1Gru Il2rgtm1Wjl Tg(HLA-DQA1,HLA-DQB1)1Dv//Sz (NSG HLA-DQ8) mice were obtained from the Jackson Laboratory. miR142-/- mice were kindly provided by S.B..<br>For experiments with NOD mice only females were used. All other experiments were performed with both sexes. Mice were aged 3 to 10 weeks when the experimental procedure began.
Wild animals	The study did not involve wild animals.
Field-collected samples	The study did not involve samples collected from the field.
Ethics oversight	District Government of Upper Bavaria, Munich, Germany (approval # ROB-55.2-2532.Vet_02-17-130).

Note that full information on the approval of the study protocol must also be provided in the manuscript.

Human research participants

Policy information about studies involving human research participants

Population characteristics	Subjects have been stratified based on the presence or absence of multiple islet autoantibodies and T1D. No islet autoimmunity and no T1D: n = 6; median age at sampling = 8 years, IQR (interquartile range) = 6-12 years; all male. Recent onset of T1D: n = 10; median age at sampling = 4 years, IQR = 3-5 years; median HbA1c = 8.9 mg/dl, IQR = 8.4-10.6 mg/dl; median time from diagnosis to sampling = 7 days, IQR = 1-11 days; all male.
Recruitment	All subjects gave written consent prior to inclusion in the Munich Bioresource project and have been already enrolled in the BABYDIAB study and the DiMelli study with the documented age of T1D onset.
Ethics oversight	Munich Bioresource project (approval number #5049/11, Technische Universität München, Munich, Germany)

Note that full information on the approval of the study protocol must also be provided in the manuscript.

Flow Cytometry

Plots

Confirm that:

- [x] The axis labels state the marker and fluorochrome used (e.g. CD4-FITC).
- [x] The axis scales are clearly visible. Include numbers along axes only for bottom left plot of group (a 'group' is an analysis of identical markers).
- [x] All plots are contour plots with outliers or pseudocolor plots.
- [x] A numerical value for number of cells or percentage (with statistics) is provided.

Methodology

Sample preparation	Human peripheral blood mononuclear cells (PBMCs) were isolated from fresh venous blood by density gradient centrifugation using Ficoll-Paque PLUS (GE Healthcare). CD4+ T cells were purified from PBMCs by Magnetic Activated Cell Sorting (MACS) using CD4 microbeads (Miltenyi Biotec) following the manufacturers protocol.<br>Murine lymph nodes and spleens were passed through 70µm cell strainers, stained with a CD4-Biotin antibody (BD Bioscience) and MACS purified using Streptavidin Microbeads (Miltenyi Biotec) following the manufacturers protocol.
Instrument	BD FACS Aria III

Software	FACSDiva software (version 6.1.3)<br>FlowJo software (version 7.6.1)
Cell population abundance	Post-sort fractions were reanalyzed with the BD FACS Aria III. Purity of all post-sort fractions was >95%.
Gating strategy	For all gating strategies we excluded doublets and dead cells.<br>murine naive T cells: CD4+CD25-CD44-<br>murine activated T cells: CD4+CD25-CD44high<br>murine Tregs: CD4+CD25+Foxp3+<br>human naive T cells: CD4+CD3+CD45RA+CD45RO-CD127+CD25-<br>human activated T cells:CD4+CD3+CD45RA-CD45RO+CD127+CD25intermediate<br>human Tregs: CD4+CD3+CD127-CD25highFoxp3high<br>FMO controls were used to define the boundries between positive and negative cell populations.

☒ Tick this box to confirm that a figure exemplifying the gating strategy is provided in the Supplementary Information.
